# Relationships between social determinants of health and healthy body composition among Aboriginal and Torres Strait Islander youth in the Next Generation: Youth Well‐being study

**DOI:** 10.1002/hpja.927

**Published:** 2024-09-30

**Authors:** Christopher D. McKay, Lina Gubhaju, Alison J. Gibberd, Bridgette J. McNamara, Rona Macniven, Grace Joshy, Aryati Yashadhana, Ted Fields, Robyn Williams, Robert Roseby, Peter Azzopardi, Emily Banks, Sandra J. Eades

**Affiliations:** ^1^ Melbourne School of Population and Global Health The University of Melbourne Melbourne Australia; ^2^ School of Population Health UNSW Sydney Australia; ^3^ Centre for Public Health Data and Policy, National Centre for Epidemiology and Population Health, College of Health & Medicine Australian National University Canberra Australia; ^4^ Centre for Primary Health Care & Equity UNSW Sydney Australia; ^5^ Curtin Medical School Curtin University Perth Australia; ^6^ Department of Respiratory Medicine Monash Children's Hospital Melbourne Australia; ^7^ Department of Paediatrics, School of Clinical Sciences Monash University Melbourne Australia; ^8^ Murdoch Children's Research Institute Melbourne Australia; ^9^ Telethon Kids Institute Perth Australia; ^10^ Present address: Yardhura Walani—National Centre for Aboriginal and Torres Strait Islander Wellbeing Research Australian National University Canberra Australia

**Keywords:** adolescent health, Australian Aboriginal and Torres Strait Islander peoples, body composition, health inequities, obesity, social determinants of health

## Abstract

**Issue Addressed:**

Little is currently known about the relationships between body composition and the social determinants of health among Aboriginal and Torres Strait Islander youth in Australia, which could help inform policy responses to address health inequities.

**Methods:**

This study aimed to explore the relationship between various social factors and healthy body mass index (BMI) and waist/height ratio (WHtR) among Aboriginal and Torres Strait Islander youth aged 16–24 years. Baseline survey data from 531 participants of the ‘Next Generation: Youth Well‐being study’ were used. Robust Poisson regression quantified associations between healthy body composition and self‐reported individual social factors (education, employment and income, government income support, food insecurity, home environment, relationship status, racism), family factors (caregiver education and employment) and area‐level factors (remoteness, socioeconomic status).

**Results:**

Healthy body composition was less common among those living in a crowded home (healthy WHtR aPR 0.67 [0.47–0.96]) and those receiving government income support (healthy BMI aPR 0.74 [0.57–0.95]). It was more common among those with tertiary educated caregivers (healthy BMI aPR 1.84 [1.30–2.61]; healthy WHtR aPR 1.41 [1.05–1.91]) and those in a serious relationship (healthy BMI aPR 1.33 [1.02–1.75]).

**Conclusions:**

Social factors at the individual and family level are associated with healthy body composition among Aboriginal and Torres Strait Islander youth.

**So What?:**

The findings of this study highlight the potential for health benefits for youth from policies and programs that address social inequities experienced by Aboriginal and Torres Strait Islander people in Australia.

## 1 INTRODUCTION

The stark health inequities seen in Australia between Aboriginal and Torres Strait Islander and non‐Indigenous populations arise from social inequities and begin to emerge in childhood.^1^ National data show that Aboriginal and Torres Strait Islander young people have experienced an increased burden from the obesity epidemic,^2^ as have young people from lower socioeconomic backgrounds.^3^ Maintaining a healthy body composition through adolescence can reduce the risk of chronic diseases, such as diabetes and cardiovascular disease, in later life.^4^ Considering more than half the Aboriginal and Torres Strait Islander population is under 25 years of age,^5^ and cardio‐metabolic diseases are a leading cause of the health gap,^6^ improvements in youth body composition have great potential to reduce health inequities. If underlying social inequities in areas like education, employment and income were to be addressed, it may be possible to close health gaps within a generation.^1^

There is limited evidence about how the social determinants of health relate to healthy body composition for Aboriginal and Torres Strait Islander youth,^7^ though some recent studies have yielded surprising results. There is consistent evidence that high body mass is more common among children and adolescents from areas with the highest socioeconomic status (SES) compared to the lowest,^8^, ^9^, ^10^ which contrasts with national trends.^11^ It is unclear if these findings reflect individual and family‐level SES, or geographic factors, or both. Evidence about associations between other potentially important social determinants like housing and racism is inconsistent or lacking.^7^ Considering important social and economic transitions that influence adult health take place during the adolescence to young adult period,^12^ there is a need to understand what social factors are most strongly associated with health indicators like body composition within the Aboriginal and Torres Strait Islander population during this time of life.

The aim of this study was to explore how individual, family, and area‐level social factors are associated with healthy body composition among Aboriginal and Torres Strait Islander youth aged 16–24 years from the ‘Next Generation: Youth Well‐being study’ (NextGen).

## 2 METHODS

### 2.1 Study participants

Full details about NextGen are available in the study protocol.^13^ Briefly, participants were eligible if they were aged between 10 and 24 years, self‐identified as Aboriginal and/or Torres Strait Islander, and resided in Western Australia (WA), New South Wales (NSW), or the Central Australia (CA) region of Northern Territory. A total of 1311 youth participants were recruited from a mix of urban, regional and remote areas from March 2018 to March 2020. Aboriginal‐led community research officer teams undertook the recruitment, utilising their community networks (including Aboriginal community centres, sporting clubs and youth centres) and youth peer recruiters. Participants had a clinical health assessment and completed a health and well‐being survey (youth survey). Parents or carers (caregivers) of all eligible participants were invited to complete a questionnaire about themselves (caregiver survey) and one for each of their participating children. Caregiver consent was required for participants under 16 years of age, with older participants consenting for themselves.

The present study included cross‐sectional baseline data for 531 youth aged 16–24 years (following 16 exclusions) and data from 164 caregivers. Youth participants were excluded if they were pregnant, did not identify as male or female (excluded due to small numbers to preserve privacy), or had an eating disorder or congenital condition that could influence body composition.

### 2.2 Data and variables

#### 2.2.1 Outcomes

Height, weight and waist circumference were measured, and body mass index (BMI) and waist/height ratio (WHtR) were calculated (see Supporting Information File S1 for a detailed protocol). BMI was categorised using World Health Organization (WHO) classifications,^14^ or International Obesity Task Force (IOTF) age‐ and sex‐specific classifications for those under 18 years of age.^15^ Responses of ‘female’ and ‘male’ to the survey question about gender were used as a proxy for sex to apply the IOTF classifications, which are designed to align with WHO categories at 18 years of age. ‘Healthy BMI’ was defined as the ‘normal’ range of these classifications (18.5–<25 kg/m^2^ for those 18 years and over). ‘Healthy WHtR’ was defined as WHtR <0.5.^16^ Categories are consistent with previous studies involving Aboriginal and Torres Strait Islander adolescents.^9^, ^17^ WHtR has been shown to be a better predictor of adiposity in children and adolescents than BMI and waist circumference.^18^ A study of Aboriginal adolescents in northern Australia found abdominal adiposity was common among those with a healthy BMI, indicating the utility of using a waist size measure in addition to BMI.^19^

#### 2.2.2 Individual factors

The other study variables were derived from survey responses (Supporting Information File S2, Table S1). Many survey questions were sourced from previous studies involving Aboriginal and Torres Strait Islander populations.^20^, ^21^, ^22^, ^23^ Individual‐level variables were self‐reported by youth and included current secondary or tertiary study (no; yes), the highest level of schooling (left school with Year 12 completed; left school with Year 10 completed; left school before Year 10; still at school), current employment and income for the past 2 weeks (unemployed; employed and <$600 [median income category among employed participants]; employed and $600 or more), receiving government income support payments (no; yes), food insecurity in the past 12 months (never/not relevant to me; rarely/sometimes; often/always), relationship status (single/dating; married/cohabiting), current living arrangement (with parents/carers; with other relatives; independent; other living arrangement), home crowding (2 or less people per bedroom; more than 2 people per bedroom), ever experienced homelessness (no; yes); ever experienced racism (no; yes) and frequency of racism experience (never; once or twice; more frequently). Homelessness and home crowding definitions are consistent with those used in national data.^24^

#### 2.2.3 Family factors

Youth were matched to the responses of their caregivers from the caregiver survey, including caregiver's highest education level (Year 10 or below; Year 12; trade/apprenticeship/certificate; diploma/degree [includes bachelor or postgraduate level]), and current employment status (never employed; currently unemployed; currently employed).

#### 2.2.4 Area‐level factors

Geographical remoteness and area‐level SES were derived from participant postcodes. Remoteness was measured by mapping participant postcodes at baseline to the Australian Bureau of Statistics Remoteness Structure 2016 (major cities; inner regional; outer regional; remote/very remote).^25^ Area‐level SES was measured using the Indigenous Relative Socioeconomic Outcomes index (IRSEO) 2016 percentile rankings,^26^ which ranks Australian Bureau of Statistics Indigenous Areas based on employment, education, income and housing information from the 2016 Census. Categories represent IRSEO rank tertile (most disadvantaged [67–100]; mid advantage [34–66]; most advantaged [0–33]).

#### 2.2.5 Covariables

Covariables included age (continuous), gender (female; male), recruitment region (WA; NSW; CA), and whether a youth participant was a parent or caregiver of children (‘parental responsibility’). The parental responsibility variable was used only in sensitivity analyses as an additional potential confounding factor. It was derived from three survey questions and includes participants who identified being a parent/carer as their main occupation, or had at least one baby born alive, or received a government parenting payment.

### 2.3 Statistical analysis

Descriptive statistics for outcome and exposure variables were calculated. The positive binary outcomes of healthy BMI and healthy WHtR were chosen for a strengths‐based approach to analysis.^27^ The BMI outcome excluded underweight participants (*n* = 37) to estimate associations with healthy BMI compared to overweight/obese. There is no established classification for ‘underweight’ according to WHtR, so all values below 0.5 were retained in the healthy category. Primary analyses were restricted to participants with outcome data, with missing exposure and covariable data imputed (see Supporting Information File S3, Tables S2 and S3).^28^, ^29^ Robust Poisson regression estimated prevalence ratios (PRs),^30^ and a generalised estimating equations framework with an exchangeable correlation structure accounted for within‐family clustering of siblings.^31^ A causal inference approach to modelling was followed, with consideration to the limitations of cross‐sectional data, including the potential for adjustment of variables on the causal pathway.^32^ Separate models were used for each exposure variable, adjusted for age, gender and recruitment region. In sensitivity analyses, additional potential confounding variables were added to models, identified a priori using subject‐matter informed directed acyclic graphs (Supporting Information File S4, Figure S1).^33^ To assess adherence to the assumption of linearity between the outcome variables and age, visual assessment and likelihood ratio tests were undertaken.^34^ Subgroup analyses stratified by living arrangement (living with caregivers; not living with caregivers) were undertaken to examine whether the effect of individual economic indicators (employment and income, government support and food insecurity) or family factors (caregiver education and employment) varied by strata. Additional sensitivity analyses included complete‐records analyses; and healthy WHtR analyses excluding participants with WHtR <0.4 (*n* = 43), consistent with a previous study's definition of low WHtR.^9^ In interpreting statistical results, consideration was given to the size of the effect, width of confidence intervals and whether they included a null association, consistency of effect estimates across the two outcome measures, and trends in effect size and direction across ordered categorical exposures. All analyses were conducted using Stata version 16.0.

## 3 RESULTS

### 3.1 Participant characteristics

Youth participant characteristics, including social factors, BMI and WHtR, and demographics for caregivers who provided data, are presented in Table 1. There was a majority of youth in younger age groups (19 years of age or younger), with a quarter still at school, and a majority were female, and recruited in WA. Most participants were not currently studying, with 22% in current paid employment (including students with jobs). A majority were receiving government income support, living with caregivers, and had experienced racism. All individual‐level exposure variables had a proportion of missing data, ranging from 12% for undertaking current secondary or tertiary study to 32% for home crowding (Table S2). There were 214 participants with caregiver data (60% missing); with most having caregivers who were unemployed and with an education below Year 10 level. Caregivers were predominately female and aged between 35 and 49 years. Among those with postcode data (*n* = 333, 37% missing), the greatest proportion were in major cities and the least in remote/very remote areas.

**TABLE 1:** Participant characteristics.

Youth characteristics (*N* = 531)	*n* (%)^a^
Age (years, median [IQR])	19.2 [17.3–21.4]^b^
Age group	
16–17 years	185 (34.8)
18–19 years	140 (26.4)
20–21 years	103 (19.4)
22–24 years	103 (19.4)
Gender	
Female	334 (62.9)
Male	197 (37.1)
Recruitment region	
Western Australia	310 (58.4)
New South Wales	164 (30.9)
Central Australia	57 (10.7)
Highest level of schooling	
Left school with Year 12 completed	128 (28.3)
Left school with Year 10 completed	141 (31.2)
Left school before Year 10	68 (15.0)
Still at school	115 (25.4)
Currently studying (secondary or tertiary)	
No	268 (57.3)
Yes	200 (42.7)
Employment and income past 2 weeks	
Unemployed	361 (78.0)
Employed and <$600	50 (10.8)
Employed and $600 or more	52 (11.2)
Government support payments	
No	163 (37.6)
Yes	271 (62.4)
Food insecurity	
Never/not relevant to me	236 (54.6)
Rarely/sometimes	156 (36.1)
Often/always	40 (9.3)
Living arrangement	
With parents/carers	273 (60.7)
With other relatives	54 (12.0)
Independent	93 (20.7)
Other living arrangement	30 (6.7)
Relationship status	
Single/dating	341 (78.2)
Cohabiting/married	95 (21.8)
Home crowding (average of people per bedroom)	
2 or less per bedroom	304 (84.2)
>2 per bedroom	57 (15.8)
Ever experienced homelessness	
No	245 (64.0)
Yes	138 (36.0)
Ever experienced racism	
No	154 (34.3)
Yes	295 (65.7)
Frequency of racism experience	
Never	154 (36.3)
Once or twice	150 (35.4)
More frequently	120 (28.3)
Caregiver educational attainment	
Year 10 or below	130 (61.0)
Year 12	34 (16.0)
Trade/apprenticeship/certificate	24 (11.3)
Diploma/degree	25 (11.7)
Caregiver employment	
Never employed	51 (24.3)
Currently unemployed	97 (46.2)
Currently employed	62 (29.5)
Area‐level SES tertile (IRSEO)	
Most disadvantaged	56 (16.8)
Middle advantage	195 (58.6)
Most advantaged	82 (24.6)
Remoteness area	
Major cities	154 (46.2)
Inner regional	119 (35.7)
Outer regional	34 (10.2)
Remote/very remote	26 (7.8)
BMI (kg/m^2^, median [IQR])	25.0 [20.9–30.3]^b^
BMI category	
Underweight	37 (9.1)
Healthy	163 (40.0)
Overweight	98 (24.0)
Obese	110 (27.0)
WHtR (median [IQR])	0.48 [0.43–0.56]^b^
WHtR category	
Elevated (≥0.5)	176 (44.1)
Healthy (<0.5)	223 (55.9)

Caregiver demographics (*N* = 164)	*n* (%)^a^
Age (years, median [IQR])	43 [37–51]^b^
Age group	
20–34 years	23 (14.7)
35–49 years	88 (56.4)
50–64 years	31 (19.9)
65 years and over	14 (9.0)
Gender	
Female	142 (86.6)
Male	22 (13.4)
Recruitment region	
Western Australia	89 (54.3)
New South Wales	56 (34.1)
Central Australia	19 (11.6)

Abbreviations: BMI, body mass index; IRSEO, Indigenous Relative Socioeconomic Outcomes; SES, socioeconomic status; WHtR, waist/height ratio.

^a^ Category frequencies may not sum to sample total due to missing data, percentages represent the category proportions among those with data for that variable.

^b^ Median and interquartile range (IQR) for numerical variable.

**FIGURE 1:**
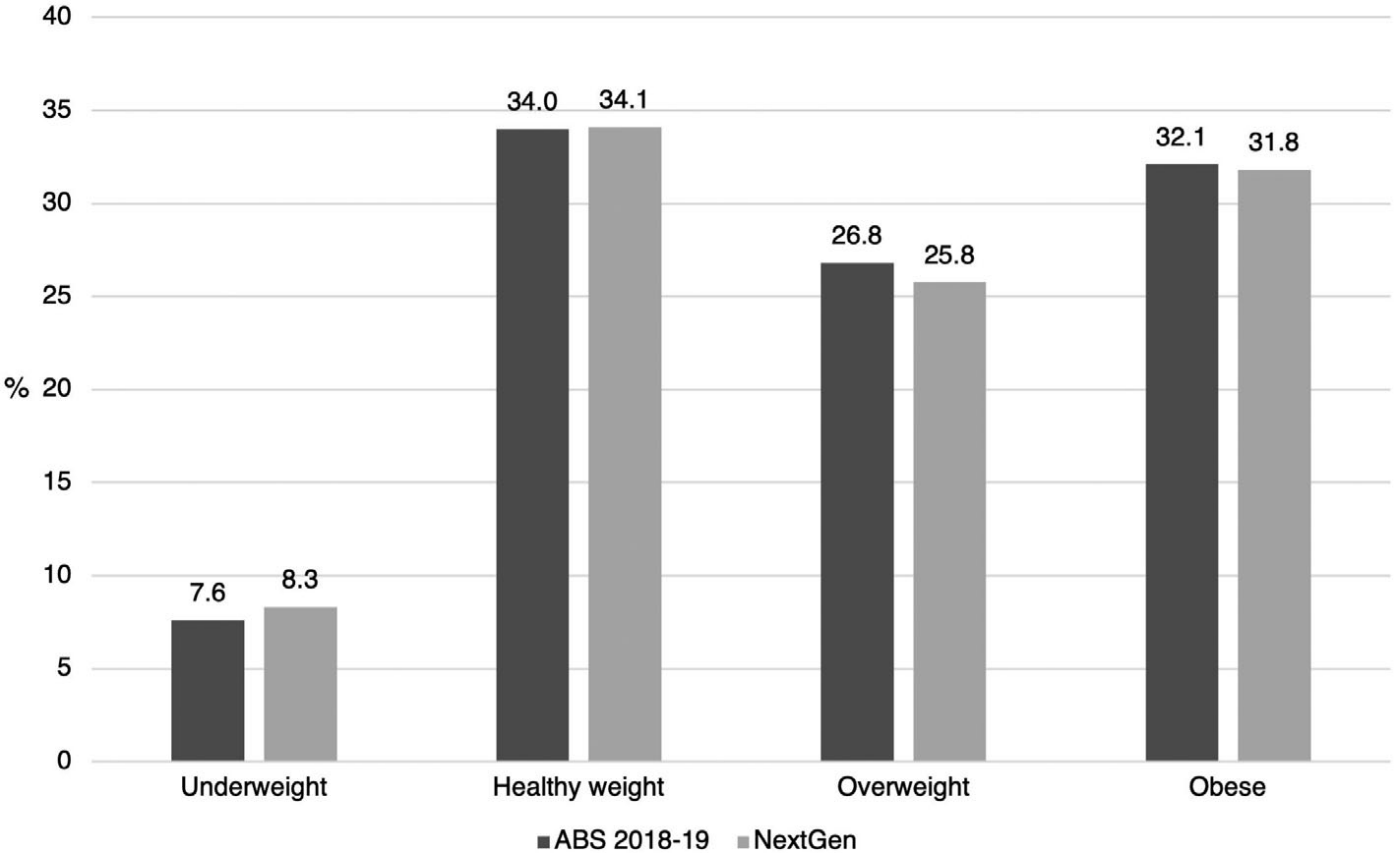
BMI category proportions for 18–24‐year olds in the National Aboriginal and Torres Strait Islander Health Survey 2018–2019 (ABS 2018–2019),^35^ and among participants with outcome data in the present study (NextGen).

There were 407 and 399 participants with BMI and WHtR data, respectively. Of those, 40% had healthy BMI and 56% had healthy WHtR. The distribution of BMI categories among 18–24‐year olds in NextGen accords with national data (Figure 1).^35^ Participants with missing outcome data were more likely to have current paid employment and diploma/degree‐educated caregivers (Table S3).

### 3.2 Associations with individual factors

**FIGURE 2:**
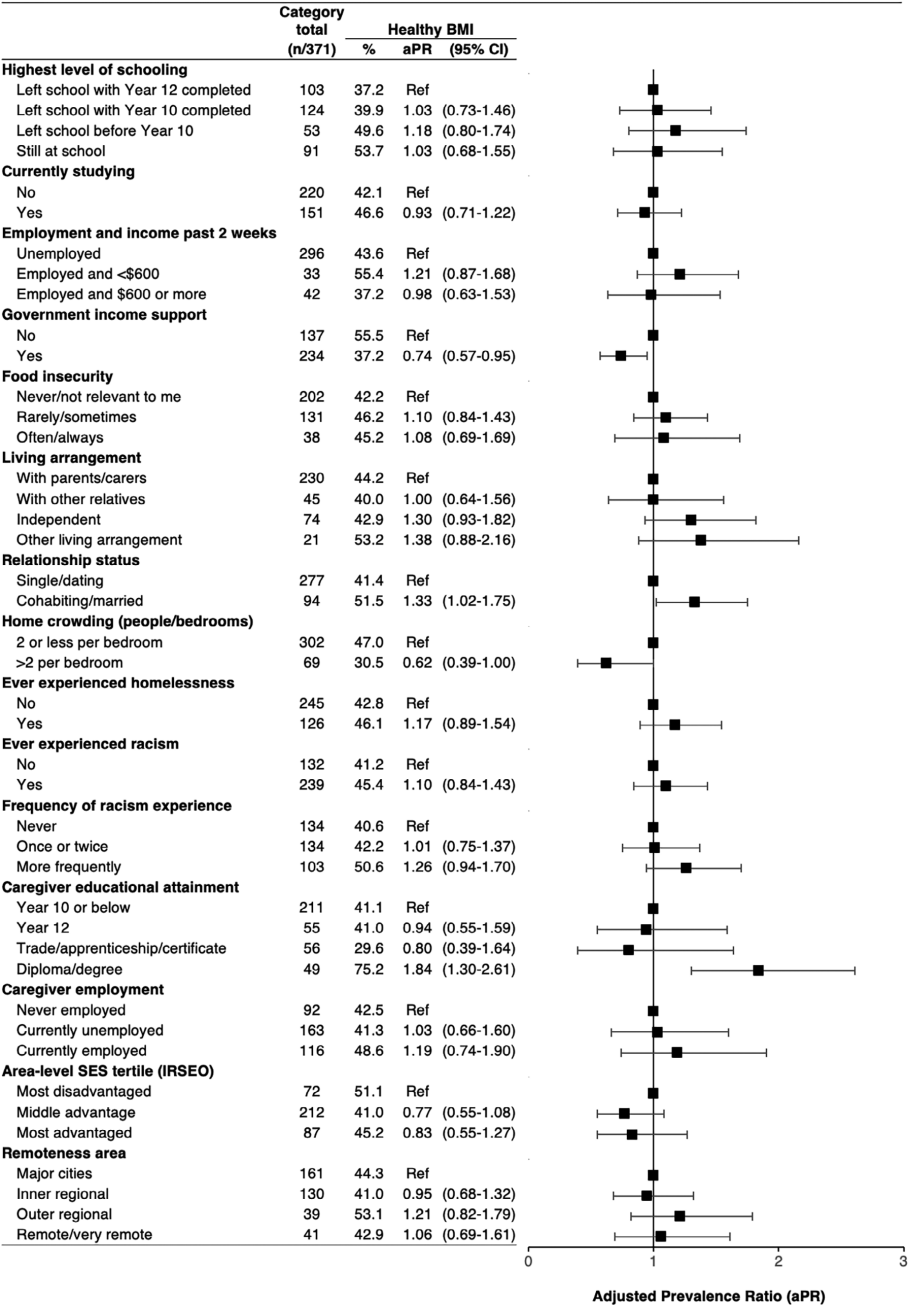
Associations between healthy body mass index (BMI) and social factors at the individual, family and area level. All statistics are pooled estimates from imputed datasets; regression models were adjusted for age, gender and recruitment region (except area‐level variables only adjusted for age and gender); plot depicts the adjusted prevalence ratios (aPR) and 95% confidence intervals, with ratios of 1.00 indicating no difference relative to reference (Ref) groups; % = unadjusted proportion with healthy BMI.

**FIGURE 3:**
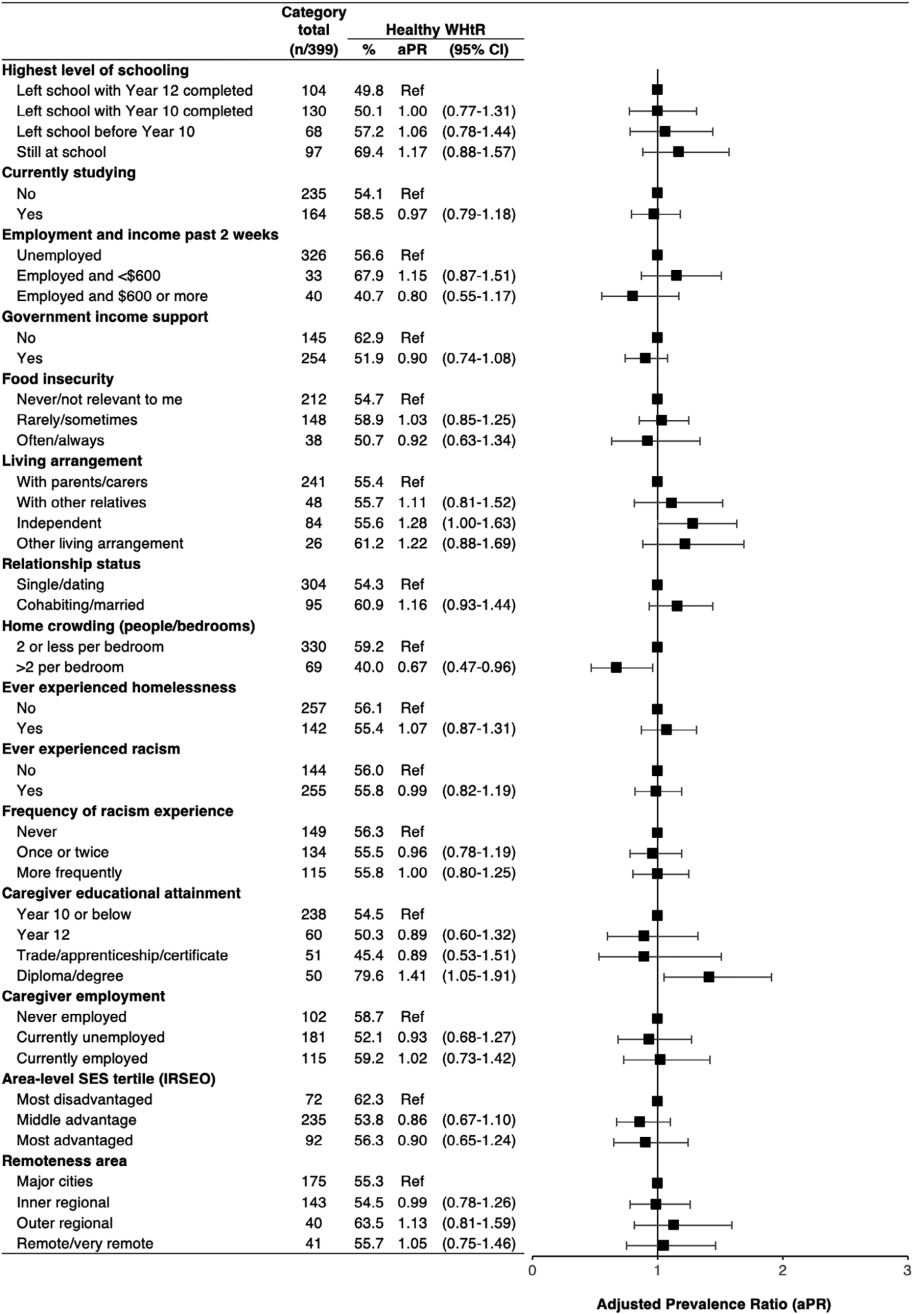
Associations between healthy waist/height ratio (WHtR) and social factors at the individual, family and area level. All statistics are pooled estimates from imputed datasets; regression models were adjusted for age, gender and recruitment region (except area‐level variables only adjusted for age and gender); plot depicts the adjusted prevalence ratios (aPR) and 95% confidence intervals, with ratios of 1.00 indicating no difference relative to reference (Ref) groups; % = unadjusted proportion with healthy WHtR.

Crude outcome proportions and adjusted PRs are presented in Figures 2 and 3. Healthy body composition was less common for participants living in crowded homes (compared to non‐crowded homes), and among participants receiving government support payments compared to those who were not. Healthy body composition outcomes were more common among those who were married or cohabiting with their partner, relative to single/dating. While crude proportions of healthy body composition outcomes were similar across categories of living arrangement, the direction of adjusted PRs suggest higher levels among youth living independently compared to youth living with caregivers. A similar effect estimate was seen for those with ‘other’ living arrangements. There was no consistent trend in the adjusted PRs by level of schooling, currently studying, employment and income, food insecurity, homelessness or racism.

### 3.3 Associations with family factors

Healthy body composition was more common among those whose caregivers had diploma/degree education compared to Year 10 or below. There was no consistent association with caregiver employment.

### 3.4 Associations with area‐level factors

There was a trend for a lower prevalence of healthy body composition among participants living in middle and most advantaged areas, compared to most disadvantaged, though the confidence intervals for all estimates included a null association. There was no clear trend by remoteness.

### 3.5 Subgroup analyses by living arrangement

Analyses stratified by living arrangement were generally consistent with results from the primary analyses (Table 2). However, estimates for the association between healthy BMI and employment and income did vary by living arrangement, with associations with opposing directions seen at the highest income level. This variation was not observed for healthy WHtR.

**TABLE 2:** Subgroup analyses stratified by living arrangement, showing associations between social factors and healthy body mass index (BMI) and waist/height ratio (WHtR).

	Healthy BMI								Healthy WHtR							
	Living with caregivers				Not living with caregivers				Living with caregivers				Not living with caregivers			
	Category total (*n*/230)	% Healthy BMI	aPR	(95% CI)	Category total (*n*/141)	% Healthy BMI	aPR	(95% CI)	Category total (*n*/241)	% Healthy WHtR	aPR	(95% CI)	Category total (*n*/158)	% Healthy WHtR	aPR	(95% CI)
Employment and income past 2 weeks																
Unemployed	189	44.8	Ref		107	41.6	Ref		199	56.3	Ref		125	57.1	Ref	
Employed and <$600	21	56	1.17	(0.78–1.74)	12	53.9	1.29	(0.65–2.56)	21	70.4	1.19	(0.86–1.64)	12	63.2	1.05	(0.61–1.81)
Employed and $600 or more	20	26.2	0.72	(0.32–1.61)	22	47.5	1.19	(0.67–2.09)	21	32.1	0.68	(0.34–1.34)	21	49.4	0.89	(0.56–1.41)
Government income support																
No	96	56.5	Ref		41	53.2	Ref		105	65	Ref		41	57.4	Ref	
Yes	134	35.3	0.68	(0.49–0.96)	100	39.7	0.84	(0.54–1.29)	136	48.1	0.82	(0.63–1.06)	117	56.3	0.99	(0.73–1.35)
Food insecurity																
Never/not relevant to me	135	43.9	Ref		66	38.5	Ref		138	55.6	Ref		73	53	Ref	
Rarely/sometimes	70	45	1.11	(0.78–1.56)	61	47.7	1.06	(0.67–1.68)	77	56.6	1.05	(0.81–1.36)	72	61.4	0.98	(0.71–1.35)
Often/always	24	42.9	1.02	(0.57–1.83)	14	49.2	1.18	(0.56–2.47)	25	50.8	0.95	(0.59–1.54)	13	50.2	0.84	(0.43–1.63)
Caregiver educational attainment																
Year 10 or below	121	41.8	Ref		90	40	Ref		130	54.7	Ref		108	54.1	Ref	
Year 12	45	40.8	0.97	(0.56–1.69)	10	40.8	0.88	(0.22–3.46)	48	49.1	0.95	(0.62–1.45)	11	53.9	0.87	(0.33–2.29)
Trade/apprenticeship/certificate	34	30.2	0.83	(0.36–1.95)	22	27.9	0.77	(0.21–2.79)	30	45.6	0.96	(0.53–1.75)	21	44.7	0.91	(0.38–2.13)
Diploma/degree	31	75.9	1.87	(1.26–2.79)	19	74.5	1.82	(0.96–3.47)	33	79.4	1.49	(1.07–2.07)	17	79.7	1.35	(0.83–2.18)
Caregiver employment																
Never employed	48	46.2	Ref		44	38.3	Ref		50	58.2	Ref		53	59.1	Ref	
Currently unemployed	98	42.6	1.00	(0.59–1.68)	65	39.2	1.14	(0.58–2.26)	107	54	0.97	(0.66–1.43)	75	49	0.93	(0.58–1.48)
Currently employed	84	45.7	1.14	(0.66–1.95)	31	57.1	1.52	(0.72–3.18)	84	56.7	1.07	(0.71–1.60)	30	66.8	1.14	(0.68–1.92)

### 3.6 Sensitivity analyses

Complete‐records analyses were consistent with the primary analyses, except the association between body composition and caregiver diploma/degree level education was attenuated in the primary analyses (Supporting Information File S5, Tables S4 and S5). In analyses where additional potential confounders were added to models and in the analyses where participants with low WHtR were excluded, results were consistent with those from the primary analyses.

## 4 DISCUSSION

This study reports novel evidence about the relationships between social factors and healthy body composition among Aboriginal and Torres Strait Islander youth in Australia. Living in a non‐crowded home and having a tertiary educated caregiver were associated with healthier body composition. There was also some evidence that not receiving government support payments, being married or cohabiting with a partner, and having an independent living arrangement were associated with healthy body composition. Unlike in previous studies, there was not a strong association between body composition and area‐level SES, though estimates were consistent with existing evidence of healthy body composition being more common in more disadvantaged areas. Together, these findings suggest that home environments have an important impact on body composition during adolescence and young adulthood and that those with higher individual SES and greater independence from caregivers have healthier body composition.

A crowded home, which reflects a lack of access to adequately sized housing, was associated with a reduced prevalence of both healthy BMI and WHtR. Only one other study has investigated this relationship among Aboriginal youth. In the Northern Territory‐based Aboriginal Birth Cohort, there was no relationship between the number of people sleeping in the home at birth, as reported by mothers, and BMI at a mean age of 25 years.^36^ The conflicting results may reflect the different exposure measures, the effect of the home environment at birth compared to the contemporary environment, or differences in the two cohorts. Aboriginal and Torres Strait Islander households tend to be larger than non‐Indigenous households, and size can fluctuate due to temporary and longer‐term visitors, driven by a culture of sharing accommodation and strong connections with extended family.^24^ Yet, there is a shortage of adequately sized public housing to accommodate this. Reducing this overcrowding through improved housing support has long been a priority issue that governments have failed to adequately address.^37^ In 2018–2019, 18% of Aboriginal and Torres Strait Islander people were living in overcrowded homes, and as high as 42% in remote areas, compared to only 5% of non‐Indigenous Australians.^38^ Overcrowding can put a strain on the resources of a household and degrade the quality of facilities, and is associated with poverty, stress and a range of other chronic health conditions.^24^ Our evidence that healthy body composition is less common among young people in crowded homes adds further weight to the urgent need for social and housing policies that consider the cultural needs of households.

Higher caregiver education was associated with healthy body composition. This was consistent with what was observed in the Longitudinal Study of Indigenous Children (LSIC), where higher maternal education was associated with lower BMI during childhood.^39^ Caregiver education level is considered a key measure of SES for children and adolescents,^40^, ^41^ and the relationship with body composition in our study is what would be expected. However, there was a high proportion of missing caregiver data, which has the potential to bias results. As missingness in the caregiver variables was associated with the outcomes (Table S3), complete‐records analysis has the potential to be biased, whereas the results from the primary analysis using imputed data may be less biased.^42^ Nevertheless, this result should be interpreted with caution.

The association between government income support payments and BMI might reflect the impact of lower individual and family SES on body composition. Access to government support in Australia is restricted by individual and household income and assets thresholds,^43^ making those with greater economic resources ineligible. These support payments are known to be below the poverty line, so individuals and families relying on them are likely to experience substantial economic difficulties and corresponding issues, such as food insecurity.^44^ Although income and assets thresholds apply to all forms of government support, there are specific factors that can influence an individual's eligibility for a particular payment, such as being a secondary or tertiary student, having children, living arrangements and relationship status. In sensitivity analyses where these factors were added to the models as potential confounding factors, the association remained and was slightly strengthened, providing greater confidence in the finding. An increase in government support payments to bring them above the poverty line, and a less punitive approach to income management and recipient obligations,^45^, ^46^ may have flow‐on benefits for the health of Aboriginal and Torres Strait Islander youth.

Our findings about relationship status and living arrangement may indicate that older adolescents and young adults with greater independence from caregivers have healthier body composition. This is particularly interesting when considering international evidence indicates BMI is more likely to increase among young adults who marry or commence a cohabiting relationship.^47^ However, there is also evidence young people with higher BMI are less likely to form relationships than those with lower BMI.^48^ The cross‐sectional nature of our study makes reverse causation a possibility. While the results of the living arrangement subgroup analyses were too imprecise to draw conclusions from, effect estimates indicated healthy BMI may be more common among those in the highest employment and income category (vs. unemployed) only when they live independently of caregivers and the opposite when they live with caregivers. Living independently of caregivers is a major event in the transition from adolescence to adulthood and is likely to have an impact on health.^12^ Future follow‐up will allow for further investigation of how such transitions influence body composition outcomes over time, what factors are associated with adolescents becoming independent earlier, and whether this independence is associated with broader wellbeing in adulthood.

Existing evidence about the impact of racism on body composition for Aboriginal and Torres Strait Islander children and youth is mixed. Our findings agree with those from the Aboriginal Birth Cohort (Northern Territory), where there was no association between self‐reported racism exposure and adiposity when participants were aged 18 years.^49^ However, in LSIC (a national sample), children who had experienced racism, as reported by their caregivers, were more likely to become obese in later childhood.^8^ Differences might result from the different measurement methods or geographic regions. Recent research involving a national sample of Aboriginal and Torres Strait Islander adults found that those who reported experiencing racial discrimination had a higher prevalence of cardio‐metabolic disease and risk factors than those with no discrimination.^50^ If racism exposure in adolescence is a determinant of cardio‐metabolic disease for adults, it may be that obesity is not the main pathway, and other risk factors like inflammation and high blood pressure may be more important.^51^ The high levels of racism exposure seen in NextGen make this a priority area of further study.

Viewed together, our results are more consistent with a conclusion that healthier body composition is associated with higher SES, in contrast to previous studies using only area‐level SES measures. We did not observe a strong association between area‐level SES and body composition in the NextGen cohort; however, the direction of the association was that children living in advantaged areas were less likely to have a healthy body composition, consistent with those previous studies.^9^, ^10^, ^36^ This creates a complex picture which may indicate there are different effects operating at the individual and area levels, and potential interactions between them. Our findings raise interesting questions about whether area‐level SES adequately reflects individual‐level SES. Previous analyses of Australian youth indicated that area‐level measures of SES substantially misclassified individual SES.^41^ Our results also contextualise recent findings of associations between body composition and cardio‐metabolic health markers and health behaviours in this population.^52^, ^53^ Future research that disentangles the different effects on body composition of individual and environmental factors, and their timing during the life course, will help inform multifactorial approaches to addressing health inequities.

SES is a multidimensional and relative concept that encompasses access to social and economic resources and occupational prestige factors.^54^ As such, composite measures of SES are often favoured for measuring youth and family SES. The most common components included in such measures are parental education and occupation, and household income.^40^, ^41^ However, for the adolescence to young adult period, it is questionable whether parental measures adequately reflect individual SES. Education, employment, income and living arrangements are all evolving during this developmental period of life, creating a measurement challenge. When using cross‐sectional data, an individual's exposure status may be too recent to have had any effect. This may explain the lack of association in our study with commonly used individual SES measures like education, employment and income. There are currently no validated composite measures of individual SES for Aboriginal and Torres Strait Islander adolescents, so it is unclear if existing methods that rely on caregiver data are culturally appropriate. From the limited caregiver data in NextGen, we see low levels of employment and educational attainment beyond Year 10, so a measure that is heavily reliant on these data may result in insufficient variation to be useful for within‐population studies. Our findings identify some potential candidate variables, such as housing factors and government income support, for alternative SES measures or inclusion in a broader, more culturally appropriate composite measure of SES. Other measures that capture the prestige element of SES in an Aboriginal and Torres Strait Islander context, like cultural roles and responsibilities, should also be explored, as well as the interaction between social and cultural determinants.

A strength of our study is that it uses data specifically collected to examine the health and wellbeing of Aboriginal and Torres Strait Islander youth from diverse regions of Australia.^13^ As such, it contributes rare data for this population group, which can inform preventive health initiatives. The findings will be particularly relevant to the communities who participated in NextGen and youth health programs in those areas. The study measured various social factors, allowing for a comprehensive exploration of associations between social determinants and body composition during adolescence, as well as an assessment of potential confounding from interrelated factors. Despite this, residual confounding may be a limitation of our results. However, our aim was not to make causal inferences from these results, for which longitudinal analyses would be required. The sample size may also have limited our capacity to detect small effects due to low statistical power. The exposures examined in this study were all self‐reported, raising the potential for measurement error. However, validity studies have shown older adolescents were able to report SES measures accurately, as compared with mothers' responses.^55^ In addition, the self‐reporting of social factors is less likely to be systematically influenced by body composition than for behavioural exposures like diet. Missing data had the potential to result in selection bias; however, we used imputation methods and compared them with complete‐records analyses to assess this possibility.

## 5 CONCLUSION

This is one of the first studies to explore the relationship between the body composition of Aboriginal and Torres Strait Islander youth and social factors at the individual, family and area levels. Through statistical analysis of a geographically diverse cohort of youth, our study provided new evidence that higher SES, as measured by factors at the individual and family level, is associated with healthy body composition. These findings provide context to previous findings of an opposite association at the area level and highlight the potential for health benefits from addressing social inequities experienced by the Aboriginal and Torres Strait Islander population.

## AUTHOR CONTRIBUTIONS

*Conceptualization*: Christopher D. McKay, Lina Gubhaju, Alison J. Gibberd, Bridgette J. McNamara and Sandra J. Eades. *Methodology*: Christopher D. McKay, Lina Gubhaju, Alison J. Gibberd, Bridgette J. McNamara and Sandra J. Eades. *Investigation*: Christopher D. McKay, Robyn Williams, Aryati Yashadhana and Ted Fields. *Formal analysis*: Christopher D. McKay. *Writing – original draft preparation*: Christopher D. McKay. *Writing – reviewing and editing*: Christopher D. McKay, Lina Gubhaju, Alison J. Gibberd, Bridgette J. McNamara, Rona Macniven, Grace Joshy, Aryati Yashadhana, Ted Fields, Robyn Williams, Robert Roseby, Peter Azzopardi, Emily Banks and Sandra J. Eades. *Funding acquisition*: Lina Gubhaju, Bridgette J. McNamara, Robert Roseby, Peter Azzopardi, Emily Banks and Sandra J. Eades. *Supervision*: Lina Gubhaju, Alison J. Gibberd, Bridgette J. McNamara and Sandra J. Eades.

## CONFLICT OF INTEREST STATEMENT

The authors declare no conflict of interest.

## ETHICS STATEMENT

Ethics approvals have been granted by the Central Australian Aboriginal Human Research Ethics Committee (16‐398), Western Australian Aboriginal Health Ethics Committee (719), Aboriginal Health and Medical Research Council of NSW Ethics Committee (1255‐17), and the University of Melbourne Medicine and Dentistry Human Ethics Sub‐Committee (1851155). NextGen is Aboriginal led, with a research team and governance committee comprised of Aboriginal and non‐Aboriginal members. Participants provided written informed consent. Consent forms advised participants that de‐identified data may be published.

## Supporting information


**Data S1.** Supporting Information.

## Data Availability

The datasets analysed during the current study are available upon reasonable request and subject to ethics approval.
