# Supplementary material for: Relationships between social determinants of health and healthy body composition among Aboriginal and Torres Strait Islander youth in the Next Generation: Youth Well‐being study
Source: Health Promot J Austr. 2024 Sep 30;36(2):e927. doi: 10.1002/hpja.927 (PMC11806405; doi:10.1002/hpja.927)
Supplement: Supplementary file 1 — Data S1. Supporting Information. [file HPJA-36-0-s001.pdf]

Supporting Information

Relationships between social determinants of health and healthy body composition among Aboriginal and Torres Strait Islander youth in the Next Generation: Youth Well-being study

Health Promotion Journal of Australia

Supplementary File 1. Protocol for measurement, calculation and data cleaning for anthropometric measures.......2

Supplementary File 2. Table of survey questions used to define exposures and covariables.......4

Supplementary File 3. Missing data assessment and multiple imputation method.......9

Supplementary File 4. Example causal diagram.......14

Supplementary File 5. Sensitivity analyses.......15

References.......19

Supplementary File 1. Protocol for measurement, calculation and data cleaning for anthropometric measures.

A clinical health assessment for youth participants was performed uniformly regardless of age by community-based researchers. The researchers attended a centralised training workshop prior to the commencement of the study to gain competency in taking clinical measures and to ensure consistency across the three study regions. Training was led by experienced clinical researchers/clinician trainers. Anthropometric measures were taken while the participant was standing on a firm surface or board, after removing shoes, belts, outer layers of clothing, and any heavy items from pockets. Weight and height were measured using a digital floor scale and stadiometer (Seca, Hamburg, Germany), to the nearest 0.1 kg and 0.5 cm, respectively. Waist circumference (WC) was measured with a tape measure (Seca, Hamburg, Germany) at the point midway between the iliac crest and the costal margin. Two measurements were recorded to the nearest 0.5 cm, and if there was a variation greater than 0.5 cm between measures one and two, a third measure was taken.

Body composition outcomes were calculated from the anthropometric measures. Height was converted to metres and then body mass index (BMI) was calculated as weight divided by the square of height (kg/m^2). Average WC (cm) was calculated and then waist/height ratio (WHtR) was calculated as average WC (cm) divided by height (cm).

Data cleaning was undertaken across the whole study cohort aged 10-24 years. We checked anthropometric data for extreme values to identify any potential data entry errors. The *zanthro* package in Stata was used to calculate age and sex-specific z-scores for height, weight and body mass index (BMI) using the UK reference ranges [1]. The UK reference ranges were used as they have an upper age limit of 23 years and comparisons with the WHO reference range yielded very similar scores. Participants who were 24 years of age were assigned z-scores using an age of 23 years. Extreme values were defined as a z-score <-5 or >5 [1]. Extreme values were retained in the dataset unless a data entry error was detected. We identified likely errors for two height values and

one weight value, and BMI for all three participants was set to missing, as was WHtR for those with height errors. A similar process was undertaken to check the waist circumference (WC) values using the UK reference range z-scores. However, as the upper age limit for this reference range was 17 years, we also considered WC values outside the range 40 to 169.5 cm to be extreme, taken from the data range for WC for participants aged 2 years and over in the US National Health and Nutrition Examination Survey 2017–2018 [2]. Following this check, four participants were identified with likely WC errors and these were set to missing.

Supplementary File 2. Table of survey questions used to define exposures and covariables.

Table S1. Study variable source questions.

**Study variable**	**Survey question(s) derived from**	**Survey question response options**<br>**(*italicised responses* were set to missing for statistical analyses, or were not used to create the study variable)**	**Study variable categories**<br>**(numbers indicate response options category derived from)**
**YOUTH SURVEY**			
Age	What is your date of birth?	Date field response	Age in years was calculated from date of birth and date of survey
Gender	Are you…	1 Female<br>2 Male<br>*Transgender female (trans woman, sistergirl)*<br>*Transgender male (trans male, brotherboy)*<br>*Other*	Female (1);<br>Male (2)<br><br>*(due to the small number of responses other than 1 or 2, the participants not identifying as female or male were unable to be included in the study to preserve privacy)*
Recruitment region	N/A – the region a participant was recruited in was encoded in their participant ID	Derived from participant ID	Western Australia;<br>New South Wales;<br>Central Australia
Currently studying (secondary or tertiary)	Which of the following BEST describes what you do most of the time:<br><br><br><br><br><br>Are you currently completing any of the following?	1 I am at school<br>2 I am at Uni/TAFE<br>3 I am working (not studying)<br>4 I am a parent/carer of a child/children (not studying or working)<br>5 Other (please specify)<br><br>6 Trade/apprenticeship<br>7 Certificate from College<br>8 Diploma (beyond Year 12)<br>9 Bachelor degree<br>10 Postgraduate diploma/higher degree<br>11 Other (please specify)	No (3,4,5*,11*);<br>Yes (1,2,6-10)<br><br>* free text responses for 'other' were checked and recategorized as 'yes' if current study was indicated.
Highest level of schooling	What is the highest level of schooling you have finished?	1 I completed primary school only<br>2 I left high school before finishing Year 10<br>3 I completed Year 10<br>4 I completed Year 12<br>5 I did not go to school	Left school with Year 12 completed (4);<br>Left school with Year 10 completed (3);<br>Left school before Year 10 (1,2,5);<br>Still at school (6)

	Which of the following BEST describes what you do most of the time:	6 I am at school<br>*I am at Uni/TAFE*<br>*I am working (not studying)*<br>*I am a parent/carer of a child/children (not studying or working)*<br>*Other (please specify)*	(free text responses for 'other' were checked and recategorized as 'still at school' if that was indicated)
Employment and income (past 2 weeks)	Do you have a job (that you get paid for) at the moment?<br><br>Which of these groupings would best describe YOUR income for the past 2 WEEKS from all sources (e.g., wages, benefits, allowances)? [only asked if responded 'Yes' to current paid employment question]	1 No<br>2 Yes<br><br>3 $1-$199<br>4 $200-$399<br>5 $400-$599<br>6 $600-$799<br>7 $800-$1999<br>8 $2000 and over<br>9 None<br>10 Other (please specify)<br>*Don't know*<br>*Prefer not to answer*	Unemployed (1);<br>Employed & <$600 (2 + 3,4,5,9,10*) ;<br>Employed & $600 or more (2 + 6,7,8)<br>* free text responses for 'other' were checked and recategorized as 'employed & $600 or more' if that was indicated.
Food insecurity (past 12 months)	In the last 12 months, HOW OFTEN have any of these happened to you, because you did not have enough of money?:<br>Went without meals /could not buy food	1 Never<br>2 Rarely<br>3 Sometimes<br>4 Often<br>5 Always<br>6 Not relevant to me	Never/not relevant to me (1,6);<br>Rarely/sometimes (2,3);<br>Often/always (4,5)
Government support payments	Do you currently receive any of the following government allowances or benefits?<br>ABstudy;<br>Youth Allowance;<br>Newstart allowance;<br>Family tax benefit (FTB) /Government Family Payment /Family Tax Benefit (FTB) as a regular payment;<br>Carer Payment /Carer Allowance; Parenting Payment;<br>Disability support pension from Centrelink;<br>Sickness Allowance;<br>Other government pension, allowance or benefit (please specify)	1 No<br>2 Yes<br>*Don't know*<br>*Prefer not to answer*	No (1);<br>Yes (2)
Living arrangement	Where do you live?	<u>Pilot survey version (n=89):</u><br>1 At home with parent(s)/carers	With parents/carers (1,8,9,10);<br>With other relatives (2,11);

		2 At a relative's place<br>3 With friends<br>4 At my own place<br>5 At an Aboriginal or Torres Strait Islander hostel<br>6 At a boarding house<br>7 Other (specify)<br><br><u>Final survey version (n=361):</u><br>8 At home with both parents/carers<br>9 At home with one parent/carer<br>10 I move between two family homes because my parents/carers do not live together<br>11 I live with other relatives<br>12 I live by myself<br>13 I live with my partner<br>14 I live in a share house /flat with other people<br>15 I live in foster care<br>16 I live in a boarding school<br>17 I live on campus at uni<br>18 I live with a friend's family<br>19 I live in a boarding house<br>20 I stay with friends/couch surf in different homes	Independent (4,12,13,14);<br>Other living arrangement (all other responses*)<br><br>* free text responses for 'other' were checked and recategorized where appropriate.
Relationship status	What is your RELATIONSHIP STATUS?	1 Single<br>2 Have a boy/girlfriend but not living together<br>3 Living with a boy/girlfriend<br>4 Married<br>5 Divorced<br>6 Separated<br>7 Widowed<br>8 Other (please specify)<br>*Prefer not to answer*	Single/dating (1,2,5,6,7,8*);<br>Cohabiting/married (3,4)<br><br>* free text responses for 'other' were checked and recategorized where appropriate.
Home crowding (average of people per bedroom)	How many bedrooms are there in your home?<br>How many ADULTS (18+) usually sleep in your home?<br>How many youths (10-17 years) usually sleep in your home?<br>How many children (0-9 years) usually sleep in your home?	Numerical responses	(Calculated as sum total of adults, youth and children that usually sleep in home divided by number of bedrooms)<br><br>2 or less per bedroom;<br>>2 per bedroom
Ever experienced homelessness	In your life have you EVER had to:<br>House hop, couch surf, staying with family or friends while you're in between houses?	1 Yes – in the past<br>2 Yes – currently<br>3 Yes – currently AND in the past	No (4);<br>Yes (1,2,3)

	Use temporary or emergency housing services (e.g., Shelter, crisis accommodation, refuges)?<br>Slept rough; outside or in an improvised dwelling, car, squatting because you had no other place to stay?	4 No<br>*Prefer not to answer*	
Ever experienced racism<br><br><br>Frequency of racism experience	Have the following EVER happened to you because you were Aboriginal and/or Torres Strait Islander?<br>You were called insulting names<br>Others left you out of their activities<br>People thought you didn't speak English well<br>Other people spat on you, pushed you or hit you<br>People acted like they were suspicious of you<br>You got poor service at a restaurant or fast food place<br>You were treated unfairly by a shop assistant or security guard	1 This did not happen to me<br>2 Once or twice<br>3 Every few weeks<br>4 About once a week<br>5 Several times a week or more	No (1);<br>Yes (2,3,4,5)<br><br>Never (1);<br>Once or twice (2);<br>More frequently (3,4,5)
Area-level SES tertile (IRSEO)	What is your postcode?	Numerical response	Most disadvantaged (67-100);<br>Middle advantage (34-66);<br>Most advantaged (0-33)<br><br>Participant postcodes were mapped to Australian Bureau of Statistics Indigenous Areas and were assigned the IRSEO 2016 rank (0-100) corresponding to the Indigenous Area accounting for the greatest proportion of the postcode area. An exception was made for CA participants living in town camps in Alice Springs, who were assigned the specific IRSEO rank formulated for these disadvantaged areas.
Remoteness area	What is your postcode?	Numerical response	Major cities;<br>Inner regional;<br>Outer regional;<br>Remote/very remote<br><br>Participant postcodes were mapped to Australian Bureau of Statistics

			Remoteness Structure 2016. As postcode areas did not perfectly match remoteness areas, the remoteness category making up the largest proportion of a postcode area was used.
Parental responsibility	Which of the following BEST describes what you do most of the time:<br><br>Have you had at least 1 baby that was born alive?<br><br><br>Do you currently receive any of the following government allowances or benefits?<br>Parenting Payment	1 I am a parent/carer of a child/children (not studying or working)<br><br>2 No<br>3 Yes<br>*Prefer not to answer*<br><br>4 No<br>5 Yes<br>*Don't know*<br>*Prefer not to answer*	No (no positive response to any of the three questions);<br>Yes (1,3,5)
**CAREGIVER SURVEY**			
Caregiver education	What is the highest level of qualifications you have achieved?	1 Year 10 or under<br>2 High school (Year 12 equivalent)<br>3 Trade/apprenticeship<br>4 Certificate from college<br>5 Diploma (beyond Year 12)<br>6 Bachelor degree<br>7 Postgraduate diploma/higher degree<br>8 Other (please specify)<br>9 None	Year 10 or below (1,8*,9);<br>Year 12 (2);<br>Trade/apprenticeship/certificate (3,4);<br>Diploma/degree (5,6,7)<br><br>* free text responses for 'other' were checked and recategorized where appropriate.
Caregiver employment	Have you ever worked in a job where you got paid?<br><br>Do you have a job (that you get paid for) at the moment?	1 No<br>2 Yes<br><br>3 No<br>4 Yes	Never (1);<br>Currently unemployed (2 + 3);<br>Currently employed (2 + 4)

Supplementary File 3. Missing data assessment and multiple imputation method.

Table S2. Study variables with proportion in missing data categories indicated (italicised).

Variable	%	(n)
**BMI**		
Underweight	7.0	(37)
Healthy	30.7	(163)
Overweight	18.5	(98)
Obese	20.5	(109)
Missing	*23.4*	*(124)*
**WHtR**		
Elevated (≥0.5)	33.1	(176)
Healthy (<0.5)	42.0	(223)
Missing	*24.9*	*(132)*
**Highest level of schooling**		
Left school with Year 12 completed	24.1	(128)
Left school with Year 10 completed	26.6	(141)
Left school before Year 10	12.8	(68)
Still at school	21.7	(115)
Missing	*14.9*	*(79)*
**Currently studying (secondary or tertiary)**		
No	50.5	(268)
Yes	37.7	(200)
Missing	*11.9*	*(63)*
**Employment and income past 2 weeks**		
Unemployed	68.0	(361)
Employed and <$600	9.4	(50)
Employed and $600 or more	9.8	(52)
Don't know/prefer not to answer	*3.0*	*(16)*
Missing	*9.8*	*(52)*
**Government support payments**		
No	30.7	(163)
Yes	51.0	(271)
Don't know/prefer not to answer	*6.6*	*(35)*
Missing	*11.7*	*(62)*
**Food insecurity**		
Never/not relevant to me	44.4	(236)
Rarely/sometimes	29.4	(156)
Often/always	7.5	(40)
Prefer not to answer	*6.4*	*(34)*
Missing	*12.2*	*(65)*
**Living arrangement**		
With parents/carers	51.4	(273)
With other relatives	10.2	(54)
Independent	17.5	(93)
Other living arrangement	5.6	(30)
Missing	*15.3*	*(81)*
**Relationship status**		
Single/dating	64.2	(341)
Cohabiting/married	17.9	(95)
Prefer not to answer	*3.8*	*(20)*
Missing	*14.1*	*(75)*
**Home crowding (average of people per bedroom)**		
2 or less per bedroom	57.3	(304)
>2 per bedroom	10.7	(57)
Missing	*32.0*	*(170)*
**Ever experienced homelessness**		
No	46.1	(245)
Yes	26.0	(138)
Prefer not to answer	*11.1*	*(59)*
Missing	*16.8*	*(89)*
**Ever experienced racism**		
No	29.0	(154)
Yes	55.6	(295)
Missing	*15.4*	*(82)*
**Parental responsibility**		
No	73.4	(390)
Yes	21.3	(113)
Missing	*5.3*	*(28)*
**Caregiver educational attainment**		
Year 10 or below	24.5	(130)
Year 12	6.4	(34)
Trade/apprenticeship/certificate	4.5	(24)
Diploma/degree	4.7	(25)
Missing	*59.9*	*(318)*

**Caregiver employment**		
Never employed	9.6	(51)
Currently unemployed	18.3	(97)
Currently employed	11.7	(62)
Missing	*60.5*	*(321)*
**Remoteness Area**		
Major cities	29.0	(154)
Inner regional	22.4	(119)
Outer regional	6.4	(34)
Remote/very remote	4.9	(26)
Missing	*37.3*	*(198)*
**Area-level SES tertile (IRSEO)**		
Most disadvantaged	10.5	(56)
Middle advantage	36.7	(195)
Most advantaged	15.4	(82)
Missing	*37.3*	*(198)*

Table S3. Relationship between analysis variables and the outcomes and outcome missingness.

	BMI								WHtR							
	Overweight/ obese		Healthy		Complete data		Missing		Elevated		Healthy		Complete data		Missing	
	%	(n)	%	(n)	%	(n)	%	(n)	%	(n)	%	(n)	%	(n)	%	(n)
**Total**	**56.1**	**(208)**	**43.9**	**(163)**	**76.8**	**(408)**	**23.2**	**(123)**	**44.1**	**(176)**	**55.9**	**(223)**	**75.1**	**(399)**	**24.9**	**(132)**
**Age group**																
16-17 years	43.4	(56)	56.6	(73)	77.8	(144)	22.2	(41)	32.1	(45)	67.9	(95)	75.7	(140)	24.3	(45)
18-19 years	60.8	(62)	39.2	(40)	82.1	(115)	17.9	(25)	47.3	(53)	52.7	(59)	80.0	(112)	20.0	(28)
20-21 years	56.9	(41)	43.1	(31)	72.8	(75)	27.2	(28)	44.6	(33)	55.4	(41)	71.8	(74)	28.2	(29)
22-24 years	71.6	(48)	28.4	(19)	70.9	(73)	29.1	(30)	61.6	(45)	38.4	(28)	70.9	(73)	29.1	(30)
**Gender**																
Female	59.4	(139)	40.6	(95)	78.4	(262)	21.6	(72)	48.2	(123)	51.8	(132)	76.3	(255)	23.7	(79)
Male	50.0	(68)	50.0	(68)	73.6	(145)	26.4	(52)	36.8	(53)	63.2	(91)	73.1	(144)	26.9	(53)
**Site**																
WA	54.8	(120)	45.2	(99)	76.8	(238)	23.2	(72)	42.9	(100)	57.1	(133)	75.2	(233)	24.8	(77)
NSW	58.6	(65)	41.4	(46)	78.0	(128)	22.0	(36)	46.8	(59)	53.2	(67)	76.8	(126)	23.2	(38)
CA	55.0	(22)	45.0	(18)	71.9	(41)	28.1	(16)	42.5	(17)	57.5	(23)	70.2	(40)	29.8	(17)
**Highest level of schooling**																
Left school with Year 12 completed	62.1	(54)	37.9	(33)	69.5	(89)	30.5	(39)	48.9	(43)	51.1	(45)	68.8	(88)	31.3	(40)
Left school with Year 10 completed	60.6	(63)	39.4	(41)	80.9	(114)	19.1	(27)	50.5	(56)	49.5	(55)	78.7	(111)	21.3	(30)
Left school before Year 10	50.0	(21)	50.0	(21)	77.9	(53)	22.1	(15)	42.6	(23)	57.4	(31)	79.4	(54)	20.6	(14)
Still at school	45.9	(34)	54.1	(40)	72.2	(83)	27.8	(32)	29.1	(23)	70.9	(56)	68.7	(79)	31.3	(36)
Missing	55.6	(35)	44.4	(28)	86.1	(68)	13.9	(11)	46.3	(31)	53.7	(36)	84.8	(67)	15.2	(12)
**Currently studying (secondary or tertiary)**																
No	57.7	(109)	42.3	(80)	77.6	(208)	22.4	(60)	46.1	(94)	53.9	(110)	76.1	(204)	23.9	(64)
Yes	53.5	(69)	46.5	(60)	71.5	(143)	28.5	(57)	40.7	(57)	59.3	(83)	70.0	(140)	30.0	(60)
Missing	55.8	(29)	44.2	(23)	88.9	(56)	11.1	(7)	45.5	(25)	54.5	(30)	87.3	(55)	12.7	(8)
**Employment and income past 2 weeks**																
Unemployed	56.2	(146)	43.8	(114)	80.1	(289)	19.9	(72)	43.7	(124)	56.3	(160)	78.7	(284)	21.3	(77)
Employed and <$600	42.9	(12)	57.1	(16)	58.0	(29)	42.0	(21)	28.6	(8)	71.4	(20)	56.0	(28)	44.0	(22)
Employed and $600 or more	60.6	(20)	39.4	(13)	65.4	(34)	34.6	(18)	58.8	(20)	41.2	(14)	65.4	(34)	34.6	(18)
Missing	59.2	(29)	40.8	(20)	80.9	(55)	19.1	(13)	45.3	(24)	54.7	(29)	77.9	(53)	22.1	(15)
**Government support payments**																
No	45.3	(48)	54.7	(58)	71.8	(117)	28.2	(46)	37.6	(44)	62.4	(73)	71.8	(117)	28.2	(46)
Yes	61.9	(117)	38.1	(72)	77.1	(209)	22.9	(62)	46.9	(97)	53.1	(110)	76.4	(207)	23.6	(64)
Missing	56.0	(42)	44.0	(33)	83.5	(81)	16.5	(16)	46.7	(35)	53.3	(40)	77.3	(75)	22.7	(22)
**Food insecurity**																
Never/ not relevant to me	60.1	(98)	39.9	(65)	75.0	(177)	25.0	(59)	45.9	(79)	54.1	(93)	72.9	(172)	27.1	(64)
Rarely/ sometimes	54.4	(56)	45.6	(47)	75.0	(117)	25.0	(39)	40.8	(49)	59.2	(71)	76.9	(120)	23.1	(36)
Often/ always	57.1	(16)	42.9	(12)	72.5	(29)	27.5	(11)	51.7	(15)	48.3	(14)	72.5	(29)	27.5	(11)
Missing	48.7	(37)	51.3	(39)	84.8	(84)	15.2	(15)	42.3	(33)	57.7	(45)	78.8	(78)	21.2	(21)
**Living arrangement**																
With parents/carers	56.3	(107)	43.7	(83)	75.1	(205)	24.9	(68)	45.4	(94)	54.6	(113)	75.8	(207)	24.2	(66)
With other relatives	61.1	(22)	38.9	(14)	77.8	(42)	22.2	(12)	46.2	(18)	53.8	(21)	72.2	(39)	27.8	(15)
Independent	59.1	(39)	40.9	(27)	77.4	(72)	22.6	(21)	46.7	(35)	53.3	(40)	80.6	(75)	19.4	(18)
Other living arrangement	50.0	(8)	50.0	(8)	70.0	(21)	30.0	(9)	40.0	(8)	60.0	(12)	66.7	(20)	33.3	(10)
Missing	50.0	(31)	50.0	(31)	82.7	(67)	17.3	(14)	36.2	(21)	63.8	(37)	71.6	(58)	28.4	(23)

**Relationship status**																
Single/dating	60.8	(138)	39.2	(89)	74.2	(253)	25.8	(88)	46.8	(117)	53.2	(133)	73.3	(250)	26.7	(91)
Cohabiting/married	50.7	(37)	49.3	(36)	81.1	(77)	18.9	(18)	41.6	(32)	58.4	(45)	81.1	(77)	18.9	(18)
Missing	45.7	(32)	54.3	(38)	81.1	(77)	18.9	(18)	37.5	(27)	62.5	(45)	75.8	(72)	24.2	(23)
**Home crowding**																
2 or less per bedroom	53.9	(110)	46.1	(94)	73.7	(224)	26.3	(80)	44.0	(99)	56.0	(126)	74.0	(225)	26.0	(79)
>2 per bedroom	70.5	(31)	29.5	(13)	78.9	(45)	21.1	(12)	64.4	(29)	35.6	(16)	78.9	(45)	21.1	(12)
Missing	54.1	(66)	45.9	(56)	81.2	(138)	18.8	(32)	37.2	(48)	62.8	(81)	75.9	(129)	24.1	(41)
**Ever experienced homelessness**																
No	59.8	(104)	40.2	(70)	76.7	(188)	23.3	(57)	45.9	(84)	54.1	(99)	74.7	(183)	25.3	(62)
Yes	55.1	(49)	44.9	(40)	71.0	(98)	29.0	(40)	47.1	(49)	52.9	(55)	75.4	(104)	24.6	(34)
Missing	50.5	(54)	49.5	(53)	81.8	(121)	18.2	(27)	38.4	(43)	61.6	(69)	75.7	(112)	24.3	(36)
**Ever experienced racism**																
No	61.8	(68)	38.2	(42)	79.9	(123)	20.1	(31)	46.3	(56)	53.7	(65)	78.6	(121)	21.4	(33)
Yes	57.3	(114)	42.7	(85)	73.6	(217)	26.4	(78)	46.5	(101)	53.5	(116)	73.6	(217)	26.4	(78)
Missing	41.0	(25)	59.0	(36)	81.7	(67)	18.3	(15)	31.1	(19)	68.9	(42)	74.4	(61)	25.6	(21)
**Caregiver educational attainment**																
Year 10 or below	61.2	(63)	38.8	(40)	90.0	(117)	10.0	(13)	51.3	(60)	48.7	(57)	90.0	(117)	10.0	(13)
Year 12	61.5	(16)	38.5	(10)	82.4	(28)	17.6	(6)	53.6	(15)	46.4	(13)	82.4	(28)	17.6	(6)
Trade/apprenticeship/certificate	71.4	(15)	28.6	(6)	87.5	(21)	12.5	(3)	57.9	(11)	42.1	(8)	79.2	(19)	20.8	(5)
Diploma/degree	22.2	(4)	77.8	(14)	72.0	(18)	28.0	(7)	21.1	(4)	78.9	(15)	76.0	(19)	24.0	(6)
Missing	54.0	(109)	46.0	(93)	70.1	(223)	29.9	(95)	39.8	(86)	60.2	(130)	67.9	(216)	32.1	(102)
**Caregiver employment**																
Never employed	58.5	(24)	41.5	(17)	94.1	(48)	5.9	(3)	45.7	(21)	54.3	(25)	90.2	(46)	9.8	(5)
Currently unemployed	60.8	(45)	39.2	(29)	84.5	(82)	15.5	(15)	52.4	(44)	47.6	(40)	86.6	(84)	13.4	(13)
Currently employed	52.0	(26)	48.0	(24)	82.3	(51)	17.7	(11)	44.0	(22)	56.0	(28)	80.6	(50)	19.4	(12)
Missing	54.6	(112)	45.4	(93)	70.4	(226)	29.6	(95)	40.6	(89)	59.4	(130)	68.2	(219)	31.8	(102)
**Area-level SES tertile (IRSEO)**																
Most disadvantaged	47.8	(22)	52.2	(24)	91.1	(51)	8.9	(5)	37.0	(17)	63.0	(29)	82.1	(46)	17.9	(10)
Middle advantage	60.6	(86)	39.4	(56)	79.0	(154)	21.0	(41)	47.8	(75)	52.2	(82)	80.5	(157)	19.5	(38)
Most advantaged	53.4	(31)	46.6	(27)	76.8	(63)	23.2	(19)	41.7	(25)	58.3	(35)	73.2	(60)	26.8	(22)
Missing	54.8	(68)	45.2	(56)	70.2	(139)	29.8	(59)	43.4	(59)	56.6	(77)	68.7	(136)	31.3	(62)
**Remoteness Area**																
Major cities	55.1	(59)	44.9	(48)	73.4	(113)	26.6	(41)	44.3	(51)	55.7	(64)	74.7	(115)	25.3	(39)
Inner regional	61.1	(55)	38.9	(35)	84.0	(100)	16.0	(19)	46.4	(45)	53.6	(52)	81.5	(97)	18.5	(22)
Outer regional	44.4	(12)	55.6	(15)	94.1	(32)	5.9	(2)	33.3	(9)	66.7	(18)	79.4	(27)	20.6	(7)
Remote/ Very remote	59.1	(13)	40.9	(9)	88.5	(23)	11.5	(3)	50.0	(12)	50.0	(12)	92.3	(24)	7.7	(2)
Missing	54.8	(68)	45.2	(56)	70.2	(139)	29.8	(59)	43.4	(59)	56.6	(77)	68.7	(136)	31.3	(62)
**Parental responsibility**																
No	54.2	(147)	45.8	(124)	76.4	(298)	23.6	(92)	42.3	(123)	57.7	(168)	74.6	(291)	25.4	(99)
Yes	63.0	(46)	37.0	(27)	71.7	(81)	28.3	(32)	51.9	(42)	48.1	(39)	71.7	(81)	28.3	(32)
Missing	53.8	(14)	46.2	(12)	100.0	(28)	0	(0)	40.7	(11)	59.3	(16)	96.4	(27)	3.6	(1)

Multiple imputation method

Multiple imputation using chained equations was used to impute missing exposure and covariable data to help reduce bias and increase the precision of estimates [3]. Separate imputation models were used for each outcome, with age, gender and recruitment region included in all models. All individual-level exposures and the parental responsibility covariable were imputed together in the same imputation model (except frequency of racism exposure, which substituted for the binary racism variable in a separate imputation model). The family factors were imputed together, with individual-level variables associated with their missingness as auxiliary variables (employment, living arrangement, and relationship status). The area-level variables were imputed separately to avoid convergence issues, along with the individual-level variables associated with postcode missingness (living arrangement and relationship status). Stata's *mi impute chained* command was used to impute 100 datasets, which were subsequently pooled according to Rubin's rules [4].

Supplementary File 4. Example causal diagram.

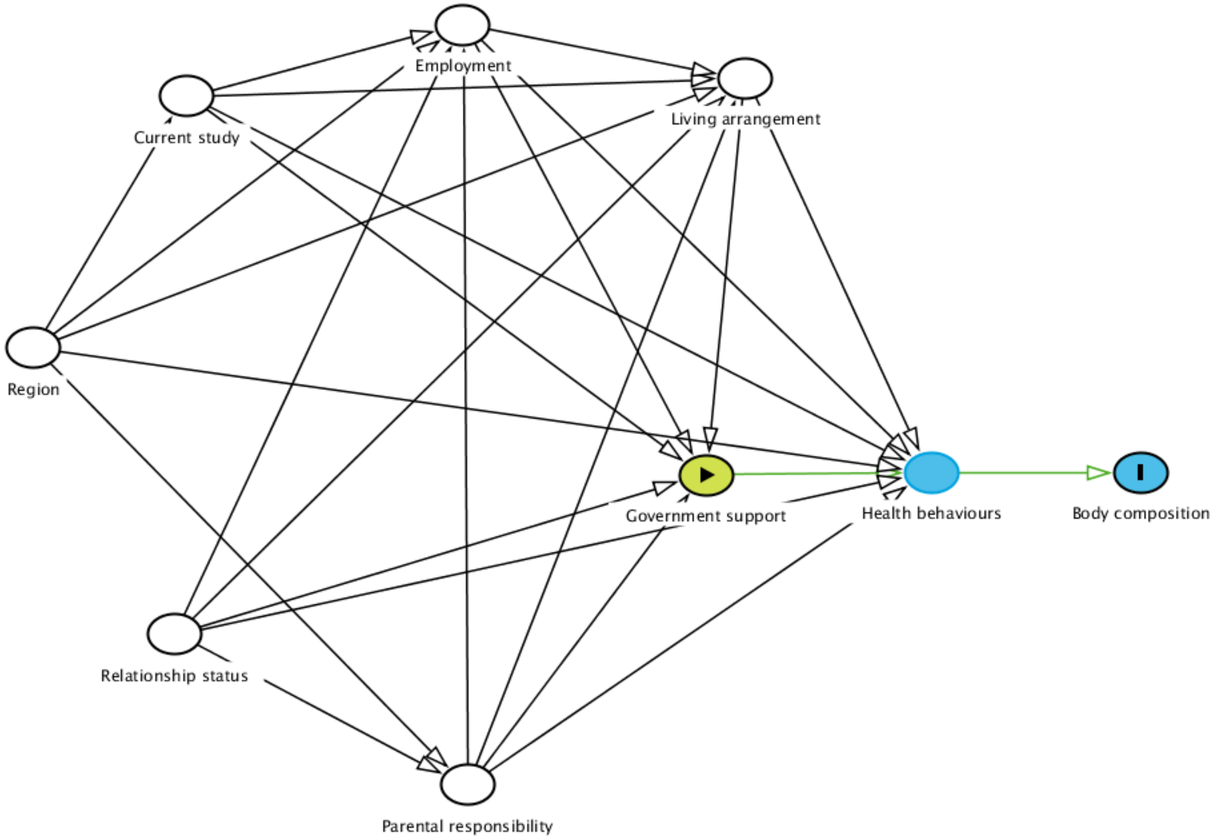

Figure S1. Causal diagram for the relationship between government income support and body composition. The model assumes government income support is a socioeconomic indicator and its relationship with body composition is mediated through health behaviours. Potential confounding variables that have been adjusted for are represented by white ovals. Age and gender were also adjusted for, though are not shown to make clearer the relationships between social factors. Image produced with DAGitty, www.daggity.net

Supplementary File 5. Sensitivity analyses.

Table S4. Comparison of estimates from the primary analyses for healthy BMI with estimates from sensitivity and complete-records analyses.

	Analyses using imputed data						Complete records analyses			
			Primary analyses		Additional confounders					
	Category total (n/371)	% healthy BMI	aPR	(95% CI)	aPR	(95% CI)	Category total (n/*)	% healthy BMI	aPR	(95% CI)
**Highest level of schooling**										
Left school with Year 12 completed	103	37.2	Ref		—	—	87	37.9	Ref	
Left school with Year 10 completed	124	39.9	1.03	(0.73-1.46)	—	—	105	39.0	1.03	(0.72-1.49)
Left school before Year 10	53	49.6	1.18	(0.80-1.74)	—	—	42	50.0	1.28	(0.87-1.88)
Still at school	91	53.7	1.03	(0.68-1.55)	—	—	74	54.1	1.00	(0.66-1.52)
**Currently studying** [a]										
No	220	42.1	Ref		Ref		190	42.1	Ref	
Yes	151	46.6	0.93	(0.71-1.22)	0.92	(0.70-1.22)	129	46.5	0.90	(0.68-1.19)
**Employment and income past 2 weeks** [b]										
Unemployed	296	43.6	Ref		Ref		260	43.8	Ref	
Employed and <$600	33	55.4	1.21	(0.87-1.68)	1.20	(0.86-1.66)	28	57.1	1.21	(0.87-1.68)
Employed and $600 or more	42	37.2	0.98	(0.63-1.53)	0.96	(0.61-1.51)	34	38.2	1.01	(0.65-1.56)
**Government income support** [c]										
No	137	55.5	Ref		Ref		107	54.2	Ref	
Yes	234	37.2	0.74	(0.57-0.95)	0.69	(0.52-0.91)	189	38.1	0.76	(0.59-0.99)
**Food insecurity** [d]										
Never/not relevant to me	202	42.2	Ref		Ref		163	39.9	Ref	
Rarely/sometimes	131	46.2	1.10	(0.84-1.43)	1.03	(0.78-1.36)	104	45.2	1.12	(0.85-1.48)
Often/always	38	45.2	1.08	(0.69-1.69)	1.00	(0.63-1.58)	28	42.9	1.09	(0.67-1.77)
**Living arrangement** [e]										
With parents/carers	230	44.2	Ref		Ref		191	43.5	Ref	
With other relatives	45	40.0	1.00	(0.64-1.56)	0.98	(0.63-1.53)	36	38.9	0.98	(0.63-1.52)
Independent	74	42.9	1.30	(0.93-1.82)	1.14	(0.75-1.72)	66	40.9	1.32	(0.93-1.88)
Other living arrangement	21	53.2	1.38	(0.88-2.16)	1.31	(0.84-2.06)	16	50.0	1.35	(0.87-2.10)
**Relationship status** [f]										
Single/dating	277	41.4	Ref		Ref		227	39.2	Ref	
Cohabiting/married	94	51.5	1.33	(1.02-1.75)	1.34	(1.02-1.76)	74	48.6	1.42	(1.06-1.88)
**Home crowding (people/bedrooms)** [g]										
2 or less per bedroom	302	47.0	Ref		Ref		205	45.9	Ref	
>2 per bedroom	69	30.5	0.62	(0.39-1.00)	0.63	(0.39-1.01)	44	29.5	0.60	(0.37-0.98)
**Ever experienced homelessness**										
No	245	42.8	Ref		—	—	174	40.2	Ref	
Yes	126	46.1	1.17	(0.89-1.54)	—	—	90	44.4	1.20	(0.90-1.62)
**Ever experienced racism**										
No	132	41.2	Ref		—	—	110	38.2	Ref	
Yes	239	45.4	1.10	(0.84-1.43)	—	—	200	42.5	1.10	(0.83-1.47)
**Frequency of racism experience**										
Never	134	40.6	Ref		—	—	110	38.2	Ref	
Once or twice	134	42.2	1.01	(0.75-1.37)	—	—	110	39.1	0.96	(0.69-1.33)
More frequently	103	50.6	1.26	(0.94-1.70)	—	—	82	47.6	1.26	(0.93-1.71)

**Caregiver educational attainment**										
Year 10 or below	211	41.1	Ref		—	—	104	38.5	Ref	
Year 12	55	41.0	0.94	(0.55-1.59)	—	—	26	38.5	0.94	(0.56-1.57)
Trade/apprenticeship/certificate	56	29.6	0.80	(0.39-1.64)	—	—	21	28.6	0.84	(0.37-1.88)
Diploma/degree	49	75.2	1.84	(1.30-2.61)	—	—	18	77.8	2.00	(1.42-2.81)
**Caregiver employment**										
Never employed	92	42.5	Ref		—	—	41	41.5	Ref	
Currently unemployed	163	41.3	1.03	(0.66-1.60)	—	—	75	38.7	1.00	(0.64-1.56)
Currently employed	116	48.6	1.19	(0.74-1.90)	—	—	50	48.0	1.24	(0.77-1.99)
**Area-level SES tertile (IRSEO)**										
Most disadvantaged	72	51.1	Ref		—	—	46	52.2	Ref	
Middle advantage	212	41.0	0.77	(0.55-1.08)	—	—	143	39.2	0.74	(0.52-1.05)
Most advantaged	87	45.2	0.83	(0.55-1.27)	—	—	58	46.6	0.88	(0.58-1.32)
**Remoteness area**										
Major cities	161	44.3	Ref		—	—	107	44.9	Ref	
Inner regional	130	41.0	0.95	(0.68-1.32)	—	—	90	38.9	0.89	(0.63-1.24)
Outer regional	39	53.1	1.21	(0.82-1.79)	—	—	27	55.6	1.22	(0.83-1.79)
Remote/very remote	41	42.9	1.06	(0.69-1.61)	—	—	23	39.1	0.97	(0.56-1.68)

All models adjusted for age, gender, and recruitment region; additional variables added to the 'additional confounders' models as per superscript: [a] employment and income, parental responsibility; [b] currently studying, parental responsibility; [c] currently studying, employment and income, living arrangement, relationship status, parental responsibility; [d] employment and income, living arrangement, relationship status, parental responsibility; [e] currently studying, employment and income, relationship status, parental responsibility; [f] parental responsibility; [g] living arrangement; * sample size varies in complete records analyses depending on missing data on the exposure variable; aPR = adjusted prevalence ratio.

Table S5. Comparison of estimates from the primary analyses for healthy WHtR with estimates from sensitivity and complete-records analyses.

	Analyses using imputed data						Imputed data excluding low WHtR (<0.4)				Complete records analyses			
			Primary analyses		Additional confounders									
	Category total (n/399)	% healthy WHtR	aPR	(95% CI)	aPR	(95% CI)	Category total (n/356)	% healthy WHtR	aPR	(95% CI)	Category total (n/*)	% healthy WHtR	aPR	(95% CI)
**Highest level of schooling**														
Left school with Year 12 completed	104	49.8	Ref		—	—	103	48.7	Ref		88	51.1	Ref	
Left school with Year 10 completed	130	50.1	1.00	(0.77-1.31)	—	—	121	45.5	0.93	(0.69-1.26)	111	49.5	0.97	(0.74-1.28)
Left school before Year 10	68	57.2	1.06	(0.78-1.44)	—	—	56	50.1	1.00	(0.71-1.40)	54	57.4	1.12	(0.83-1.52)
Still at school	97	69.4	1.17	(0.88-1.57)	—	—	76	61.4	1.00	(0.70-1.43)	79	70.9	1.16	(0.85-1.57)
**Currently studying** [a]														
No	235	54.1	Ref		Ref		216	49.7	Ref		204	53.9	Ref	
Yes	164	58.5	0.97	(0.79-1.18)	0.94	(0.76-1.15)	140	51.9	0.92	(0.73-1.17)	140	59.3	0.96	(0.78-1.18)
**Employment and income past 2 weeks** [b]														
Unemployed	326	56.6	Ref		Ref		287	50.8	Ref		284	56.3	Ref	
Employed and <$600	33	67.9	1.15	(0.87-1.51)	1.13	(0.86-1.49)	30	65.2	1.23	(0.91-1.65)	28	71.4	1.20	(0.93-1.54)
Employed and $600 or more	40	40.7	0.80	(0.55-1.17)	0.78	(0.53-1.16)	39	37.6	0.79	(0.51-1.23)	34	41.2	0.81	(0.55-1.19)
**Government income support** [c]														
No	145	62.9	Ref		Ref		128	57.6	Ref		117	62.4	Ref	
Yes	254	51.9	0.90	(0.74-1.08)	0.84	(0.68-1.04)	228	46.6	0.89	(0.71-1.11)	207	53.1	0.92	(0.76-1.11)
**Food insecurity** [d]														
Never/not relevant to me	212	54.7	Ref		Ref		185	48.2	Ref		172	54.1	Ref	
Rarely/sometimes	148	58.9	1.03	(0.85-1.25)	0.99	(0.81-1.20)	134	54.3	1.13	(0.90-1.43)	120	59.2	1.05	(0.86-1.27)
Often/always	38	50.7	0.92	(0.63-1.34)	0.88	(0.60-1.28)	37	48.9	1.03	(0.69-1.55)	29	48.3	0.92	(0.62-1.37)
**Living arrangement** [e]														
With parents/carers	241	55.4	Ref		Ref		217	50.5	Ref		207	54.6	Ref	
With other relatives	48	55.7	1.11	(0.81-1.52)	1.09	(0.79-1.49)	42	48.9	1.08	(0.75-1.56)	39	53.8	1.08	(0.79-1.49)
Independent	84	55.6	1.28	(1.00-1.63)	1.26	(0.94-1.69)	76	51.2	1.25	(0.94-1.66)	75	53.3	1.27	(0.97-1.66)
Other living arrangement	26	61.2	1.22	(0.88-1.69)	1.19	(0.85-1.65)	21	52.1	1.20	(0.76-1.89)	20	60.0	1.25	(0.89-1.75)
**Relationship status** [f]														
Single/dating	304	54.3	Ref		Ref		270	48.4	Ref		250	53.2	Ref	
Cohabiting/married	95	60.9	1.16	(0.93-1.44)	1.16	(0.93-1.44)	86	57.2	1.24	(0.97-1.59)	77	58.4	1.20	(0.96-1.51)
**Home crowding (people/bedrooms)** [g]														
2 or less per bedroom	330	59.2	Ref		Ref		291	53.4	Ref		225	56.0	Ref	
>2 per bedroom	69	40.0	0.67	(0.47-0.96)	0.68	(0.47-0.98)	65	37.7	0.69	(0.46-1.04)	45	35.6	0.62	(0.41-0.94)
**Ever experienced homelessness**														
No	257	56.1	Ref		—	—	228	50.9	Ref		183	54.1	Ref	
Yes	142	55.4	1.07	(0.87-1.31)	—	—	128	49.9	1.07	(0.83-1.36)	104	52.9	1.06	(0.85-1.33)
**Ever experienced racism**														
No	144	56.0	Ref		—	—	125	49.4	Ref		121	53.7	Ref	
Yes	255	55.8	0.99	(0.82-1.19)	—	—	231	51.2	1.03	(0.81-1.30)	217	53.5	0.98	(0.79-1.20)
**Frequency of racism experience**														
Never	149	56.3	Ref		—	—	129	49.3	Ref		121	53.7	Ref	
Once or twice	134	55.5	0.96	(0.78-1.19)	—	—	122	51.1	1.02	(0.78-1.34)	111	53.2	0.94	(0.75-1.18)
More frequently	115	55.8	1.00	(0.80-1.25)	—	—	105	51.4	1.05	(0.80-1.38)	94	52.1	0.98	(0.77-1.25)
**Caregiver educational attainment**														
Year 10 or below	238	54.5	Ref		—	—	207	48.4	Ref		117	48.7	Ref	
Year 12	60	50.3	0.89	(0.60-1.32)	—	—	55	44.4	0.90	(0.57-1.43)	28	46.4	0.92	(0.62-1.38)
Trade/apprenticeship/certificate	51	45.4	0.89	(0.53-1.51)	—	—	52	43.4	1.01	(0.60-1.69)	19	42.1	0.93	(0.54-1.59)
Diploma/degree	50	79.6	1.41	(1.05-1.91)	—	—	42	77.3	1.57	(1.12-2.21)	19	78.9	1.55	(1.06-2.25)

**Caregiver employment**														
Never employed	102	58.7	Ref		—	—	85	51.4	Ref		46	54.3	Ref	
Currently unemployed	181	52.1	0.93	(0.68-1.27)	—	—	167	47.9	0.97	(0.65-1.45)	84	47.6	0.93	(0.66-1.31)
Currently employed	115	59.2	1.02	(0.73-1.42)	—	—	104	53.8	1.08	(0.71-1.64)	50	56.0	1.04	(0.72-1.52)
**Area-level SES tertile (IRSEO)**														
Most disadvantaged	72	62.3	Ref		—	—	60	54.5	Ref		46	63.0	Ref	
Middle advantage	235	53.8	0.86	(0.67-1.10)	—	—	216	49.2	0.88	(0.65-1.19)	157	52.2	0.83	(0.63-1.08)
Most advantaged	92	56.3	0.90	(0.65-1.24)	—	—	81	51.3	0.90	(0.62-1.29)	60	58.3	0.94	(0.69-1.29)
**Remoteness area**														
Major cities	175	55.3	Ref		—	—	154	49.4	Ref		115	55.7	Ref	
Inner regional	143	54.5	0.99	(0.78-1.26)	—	—	134	50.9	1.05	(0.80-1.36)	97	53.6	0.95	(0.74-1.23)
Outer regional	40	63.5	1.13	(0.81-1.59)	—	—	36	59.7	1.19	(0.82-1.71)	27	66.7	1.19	(0.88-1.61)
Remote/very remote	41	55.7	1.05	(0.75-1.46)	—	—	33	44.5	0.93	(0.61-1.40)	24	50.0	0.92	(0.61-1.40)

All models adjusted for age, gender, and recruitment region; additional variables added to the 'additional confounders' models as per superscript: [a] employment and income, parental responsibility; [b] currently studying, parental responsibility; [c] currently studying, employment and income, living arrangement, relationship status, parental responsibility; [d] employment and income, living arrangement, relationship status, parental responsibility; [e] currently studying, employment and income, relationship status, parental responsibility; [f] parental responsibility; [g] living arrangement; * sample size varies in complete records analyses depending on missing data on the exposure variable; aPR = adjusted prevalence ratio.

References

1. Vidmar, S. I., Cole, T. J., & Pan, H. (2013). Standardizing anthropometric measures in children and adolescents with functions for egen: Update. *The Stata Journal, 13*(2), 366-378.

2. Centers for Disease Control and Prevention. (2020, 2020). *National Health and Nutrition Examination Survey: 2017-2018 Data Documentation, Codebook, and Frequencies*. CDC. Retrieved 5 September 2022 from https://wwwn.cdc.gov/Nchs/Nhanes/2017-2018/BMX_J.htm

3. White, I. R., Royston, P., & Wood, A. M. (2011). Multiple imputation using chained equations: issues and guidance for practice. *Statistics in medicine, 30*(4), 377-399. https://doi.org/10.1002/sim.4067

4. Rubin, D. B. (1987). *Multiple Imputation for Nonresponse in Surveys*. Wiley.
